# 

**Published:** 1891-08-01

**Authors:** 


In the "Lancet" of Nov. 11, 1865, BARON LIEBIG says :—" Were it possible to furnish the market at a reasonable price with a preparation of meat, combining in itself the albuminous together with the extractive principles, such a preparation would have to be preferred to the Extractum Carnis, for it would contain all the nutritive constituents of meat." Again—"I have before stated that in pre- [illegible] paring the Extract of Meat, the Albuminous principles remain in the residue, they are [illegible] ost to nutrition, and this is certainly a great disadvantage." "BOVRIL" contains [illegible] he albumen and fibrine in the most perfect possible form, and to [illegible] know the requirements of the human system and the [illegible] onstituents of food, it will be apparent that this albumen and [illegible] identical with what the body requires for recuperation, [illegible] and that as a perfect form of concentrated nourishment it must [illegible] any animal aliment at present known.

SOLD THROUGHOUT THE UNITED KINGDOM.

### THE HOSPITAL

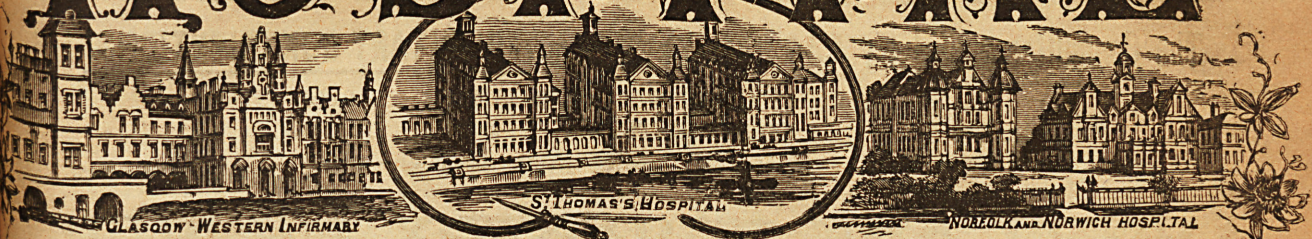

WITH EXTRA NURSING SUPPLEMENT.

No. 253. Vol. X.] SATURDAY, AUGUST 1st, 1891. [ONE PENNY.

